# TBT Effects on the Development of Intersex (Ovotestis) in Female Fresh Water Prawn *Macrobrachium rosenbergii*

**DOI:** 10.1155/2014/412619

**Published:** 2014-07-10

**Authors:** Revathi Peranandam, Iyapparaj Palanisamy, Arockia Vasanthi Lourdaraj, Munuswamy Natesan, Arun Prasanna Vimalananthan, Suganya Thangaiyan, Anantharaman Perumal, Krishnan Muthukalingan

**Affiliations:** ^1^Department of Environmental Biotechnology, Bharathidasan University, Trichy, Tamil Nadu 620 024, India; ^2^CAS in Marine Biology, Faculty of Marine Sciences, Annamalai University, Parangipettai, Tamil Nadu 608 502, India; ^3^Department of Zoology, University of Madras, Guindy Campus, Chennai, Tamil Nadu 600 025, India

## Abstract

The impact of tributyltin (TBT) on the female gonad and the endocrine system in *Macrobrachium rosenbergii* was studied. Prawns were exposed to environmentally realistic concentrations of 10, 100, and 1000 ng/L of TBT for 6 months. Dose dependent effects were noticed in TBT exposed prawns. At 1000 ng/L TBT caused ovotestis formation (formation of male germ cells in ovary). Presence immature oocytes, fusion of developing oocytes, increase in interstitial connective tissues, and its modification into tubular like structure and abundance of spermatogonia in the ovary of TBT treated prawns. The control prawn ovary showed normal architecture of cellular organelles such as mature oocytes with type 2 yolk globules, lipid droplets, normal appearance of yolk envelop, and uniformly arranged microvilli. On the other hand, type 1 yolk globules, reduced size of microvilli, spermatogonial cells in ovary, spermatogonia with centrally located nucleus, and chromatin distribution throughout the nucleoplasm were present in the TBT treated group. Immunofluorescence staining indicated a reduction in vitellin content in ovary of TBT treated prawn. Moreover, TBT had inhibited the vitellogenesis by causing hormonal imbalance in *M. rosenbergii*. Thus, the present investigation demonstrates that TBT substantially affects sexual differentiation and gonadal development in *M. rosenbergii*.

## 1. Introduction

It is now well established that various natural and man-made compounds in domestic and industrial effluents are capable of altering reproductive processes in wildlife [1]. The list of endocrine disrupting chemicals (EDCs) includes octylphenol (OP) and nonylphenol (NP), phthalates, chlorotriazines, dioxins, poly aromatic hydrocarbons (PAHs), poly chlorinated biphenols (PCBs), polychlorinated dibenzodioxins (PCDDs), polychlorinated dibenzofurans (PCDFs) and various pesticides [2]. EDCs have the potential to mimic the action of natural hormones by binding to their receptors or to modulate or disrupt the synthesis, secretion, transport, binding, or elimination of endogenous hormones in the body and consequently to affect homeostasis, development, reproduction, and behavior of organisms [3, 4].

Organotin compounds, particularly tributyltin (TBT), have been reported to be strong endocrine disrupting compounds (EDC). TBT is highly toxic to many aquatic organisms and is still detected in aquatic environments though it had been banned in antifouling paints and as biocides in a variety of consumer and industrial products [5]. The level of TBT in the aquatic environment is still a cause of great concern [6]. Effects of TBT have been investigated in several aquatic organisms, including algae [7] and crustaceans [8]. The administration of TBT adversely affects the growth and reproductive activity in* Perna indica* [9]. For instance, our previous study revealed the marked inhibition in organogenesis [10], spermatogenesis [11, 12], and vitellogenesis in* M. rosenbergii* [5].

Hormones have a critical function in the regulation of internal homeostasis in an organism [13]. Some of the natural and xenoestrogenic compounds have adversely affect the development, differentiation, and reproduction of aquatic invertebrates like coelenterate* Hydra vulgaris, *insect* Chironomus riparius*, crustaceans* Hyalella azteca, Gammarus pulex,* and mollusc* Lymnaea stagnalis *[13].

Impaired sexual development by TBT is well known in molluscs with imposex as the best-documented example [14]. Disturbances in the secretion of neuroendocrinological factors [15, 16] and hormonal disruption by inhibition of biotransformation enzymes [17] have been proposed as potential causes for the development of imposex. Nishikawa et al. [18] reported that TBT is a strong agonist of retinoid X receptor and induced imposex in molluscs via this pathway, whereas imposex has also been shown to be caused by a TBT induced inhibition of aromatase in molluscs [19]. The prevailing biochemical hypotheses for the induction of imposex comprise these two major pathways [20, 21].

The synthesis of vitellogenin is under control of estrogen, and this protein is acknowledged as a good biomarker of estrogenic exposure in aquatic organisms [22, 23]. Histopathological examinations may provide insight into the nature of reproductive impairments [24–27]. Besides, ultrastructural study is recognized as an important tool to evaluate the effects of contaminants on vital organs, which will ultimately affect various other metabolic processes such as growth and reproduction. This tool is also helpful in detecting early effects of toxicants on cells [28].

Laboratory studies are now helping to determine the potency of these various EDCs and their potential threats to the aquatic environment [29, 30]. Stentiford and Feist [31] stated that little researches have been done with estuarine organisms. Most studies investigating endocrine disruption in the aquatic environment that have focused on vertebrates have found less attention despite the fact that they constitute 95% of all living animal species and play an essential role in the functioning and health of aquatic ecosystems [32].

There is extensive evidence for the adverse impact of TBT in molluscs which led to imposex phenomenon. Contrastingly, only a few articles have addressed the deleterious effects of TBT in crustaceans [12]. Especially with reference to TBT toxicity in commercially important fresh water prawn* M. rosenbergii*, not much information is available [10]. Hence, the present work was made to explore the TBT induced masculinization in* M. rosenbergii*. The objective of the present study is, therefore, to analyse the effects of TBT on structural changes in the ovary, assessment of reproductive biomarker, and evaluation of sex hormonal balance in* Macrobrachium rosenbergii*.

## 2. Material and Methods

### 2.1. Collection and Maintenance of Prawn

Freshwater female prawns,* M. rosenbergii,* were collected from the Aqua Nova hatchery in Kanathur near Chennai, South India. The collected prawns were brought to the laboratory in a plastic cover with habitat water. They were introduced into plastic tanks with sufficient aeration. The water was changed daily and prawns were fed* ad libitum* with commercial pelletized feed. They were maintained in the laboratory for 2-3 weeks for acclimatization.

### 2.2. Experimental Design and TBT Treatment

Five-month-old prawns (75 individuals weighing 16 ± 2 g/each) were selected and divided into 5 groups (15 individuals/group). The first group served as control (without any treatment). As ethanol is a solvent used to prepare the TBT (TBT chloride) solutions, the second group served as positive control that received 2% ethanol treatment. The remaining three groups were individually exposed to environmentally realistic concentrations of TBT, that is, 10, 100, and 1000 ng/L, using water as the medium (data not shown). Each group of prawns was maintained in the individual plastic tanks containing 100 L of well-aerated water and the water subjected to static renewal. Every day, the water was exchanged and the nominal concentrations of TBT were maintained in the respective experimental tanks. For each treatment, triplicates were maintained and the experiment was conducted for a period of six months with the water temperature of 18 ± 2°C. Prawns were fed* ad libitum* with commercial pellet feed during the experiment. The bioavailability of TBT was confirmed periodically in experimental tanks.

### 2.3. Assessment of Reproductive Activity

At the end of the experiment, the prawns were weighed, gonads removed and the weight of the gonads was recorded. The gonadosomatic index (GSI) and hepatosomatic index (HSI) were calculated following the procedure outlined by Zhang et al. [33].

### 2.4. Histology

Triplicate histological analyses were done by sacrificing three animals from each group. For this, ovary was dissected out carefully. The tissue samples were fixed in Bouin's fixative for 24 h and washed with distilled water. The samples were dehydrated with different graded alcohol series and processed by routine procedure. Sections of 6–8 *μ*m thickness were made and stained with haematoxyline and eosin. The stained sections were mounted using Dibutyl phthalate xylene (DPX) and photomicrographs of varying magnifications were taken using Leica 2500 microscope.

### 2.5. Measurement of Oocyte Development

Oocyte diameter was measured using an ocular micrometer calibrated with a stage micrometer fitted in a light microscope (Labex, India). For each prawn, the diameter of at least 30 oocytes was measured and the mean oocyte diameter was calculated. The stage of oocyte development was characterized based on the maximum number of oocytes confined to a particular stage of development. Photomicrographs of various stages of oocyte development were taken using Leica 2500 microscope (Germany).

### 2.6. Transmission Electron Microscopy

For transmission electron microscopic study, three prawns were sacrificed and their ovary samples were dissected out. Then the samples were cut into small pieces and fixed in 6% glutaraldehyde prepared in 0.1 M phosphate buffer (pH 7.3) for 2–4 h. Tissues were washed in 0.1 M phosphate buffer and postfixed in 0.5% osmium tetroxide for 30 min. The samples were washed again in phosphate buffer, rapidly dehydrated in a graded alcohol series, and embedded in a low viscous epoxy-resin [34]. Ultrathin sections were cut with a diamond knife on a Sorvall MT-2B ultramicrotome and mounted on copper grids. The ultrathin sections were stained with uranyl acetate and lead citrate and analysed under a Hitachi (HU-11E2) transmission electron microscope.

### 2.7. Identification and Quantification of Vitellin and Vitellogenin

#### 2.7.1. Immunofluorescence

For immunofluorescence study, ovaries of control and TBT exposed prawns were fixed in 4% paraformaldehyde in phosphate buffer saline (PBS) (pH 7.0) at 4°C overnight. After washing with PBS (pH 7.0) three times, the samples were immersed in 30% saccharose-PBS buffer overnight at 4°C. They were then embedded individually in wax and sectioned at 6-7 *μ*m thickness using microtome (Leica). Then the sections were dehydrated in PBS for 30 min and incubated for 1 h with 5% dry milk in PBS at room temperature to prevent nonspecific binding of antibodies. The sections were then incubated overnight at 4°C with the specific primary antibody (rabbit antibody) for vitellogenin (1 : 2000 dilution). The slides were washed with PBS, subsequently incubated for 1 h with fluorescein iso thiocyanate (FITC) conjugated secondary antibody (anti-rabbit IgG, 1 : 100 dilution) in the dark, and washed five times with PBS (10 min each). Then the sections were stained by propidium iodide (PI) for 5 min and washed four times (5 min each). Finally, the sections were observed under Leica confocal fluorescence microscope.

#### 2.7.2. Isolation of Vitellogenin and Vitellin

Vitellogenin and vitellin were isolated from the hepatopancreas, hemolymph, and ovaries of prawn* M. rosenbergii* following the method of Tsukimura et al. [35]. In brief, the reproductive tissues were homogenized in homogenization buffer (containing 0.1 M NaCl, 0.05 M Tris, 1 mM ethylenediaminetetraacetic acid and 0.1% Tween 20 with 10 mg/mL PMSF; pH 7.8) using an ice cold glass homogenizer. The homogenate was centrifuged at 4000 ×g for 5 min at 4°C. The resultant supernatant was again centrifuged at 20,000 ×g for 20 min at 4°C. To the supernatant, saturated ammonium sulphate was added to produce 25% SAS solution. After incubation for 1 h at 4°C, the solution was centrifuged at 20,000 ×g for 10 min at 4°C. The supernatant was collected and saturated ammonium sulphate was added to produce 40%, 50%, and 60% saturated ammonium sulphate solution sequentially. The pellets of 60% saturated ammonium sulphate solution were suspended in appropriate volume of homogenization buffer and dialyzed thrice at 4°C for 12 h each against homogenization buffer. Further, the isolated vitellogenin and vitellin were purified by following the scheme of Zagalsky et al. [36]. Then the purified vitellogenin and vitellin were stored at −20°C until further analysis.

#### 2.7.3. Enzyme Linked Immunosorbent Assay

Hundred milligrams of hepatopancreas, ovary, and hemolymph samples was taken individually from control and TBT treated groups. Tissues were individually homogenized with phosphate buffer and centrifuged at 13,000 ×g for 10 min at 10°C to remove cellular debris. The supernatant was collected in separate vials and stored at −20°C until assay. Microtiter plates were filled with 100 *μ*L (six replicates) of different samples separately, diluted with coating buffer, and incubated over night at 4°C. After three washings with buffer, the wells were blocked with 200 *μ*L of blocking buffer and incubated at 37°C for 1 h. Washing was followed by the addition of 100 *μ*L of primary antibody (anti-Vg at 1 : 2000), for 3 h at 37°C. The primary antibody was priorly raised in rabbit using the purified Vg from* M. rosenbergii*. After three times washing, the wells were coated with 100 *μ*L secondary-antibody enzyme conjugated (anti-rabbit IgG-Alkaline phosphatase) at 1 : 500 dilutions for 1 h at 37°C. Incubation was terminated by washing and wells were filled with 100 *μ*L of substrate solution (1 mg p-Nitrophenyl Phosphate/mL of substrate buffer). The reaction was stopped with the stop buffer after the required colour development was attained. Concentrations of Vg standard ranged from 0.1 to 100 *μ*g/mL. Absorbance at 405 nm was measured in an automated ELISA plate reader (Titertek Multiskan Plus, MK II, Denmark).

### 2.8. Hormonal Assay

#### 2.8.1. Radioimmunoassay

Steroid hormone measurements were performed using radioimmunoassays (RIAs) as it is highly rapid, specific, and accurate, especially for 17*β*-estradiol and testosterone. The ovary, hemolymph, and hepatopancreas samples were collected from control and experimental prawns, shock frozen in liquid nitrogen, and homogenized individually on ice in 100 *μ*L deionized water using a motor driven Teflon pestle. Steroid metabolites were extracted using 4 mL ethyl acetate (2 × 2 mL) and the organic phase was separated using centrifugation at 5000 ×g for 15 min at 4°C. The ethyl acetate fractions were pooled and evaporated under a stream of nitrogen. The steroid extracts were individually estimated for the level of free immunoreactive 17*β*-estradiol and testosterone using radioimmunoassay (RIA) according to the protocol of Oreczyk et al. [37]. The steroid extracts (six replicates/each sample) were reconstituted separately in 100 *μ*L of gelatin phosphate buffer solution (GPBS) (sodium phosphate buffer 0.1 M, pH 7.2, containing 0.15 M NaCl and 0.1% gelatin) in RIA tubes. Appropriately diluted antiserum to 17*β*-estradiol and testosterone (New England Nuclear Corp., Boston, MA) and 0.1 mL of [3H]-steroids without antiserum (to determine nonspecific binding) were included in every assay. At the end of incubation, bound and free steroids were separated by adding 0.3 mL of dextran coated charcoal (0.1% dextran T70 and 1% charcoal in PSB) and each tube was centrifuged at 3000 ×g for 20 min at 4°C. The supernatant was poured carefully without disturbing the charcoal pellet into the vials containing 5 mL of scintillation fluid (0.5% PPO, 0.04% POPOP, and 25% methanol I toluene). The vials were shaken at room temperature to extract steroids into aqueous phase and steroid levels were estimated using a liquid scintillation counter (Beckman, USA). The NHANES (National Health and Nutrition Examination Survey, U.S) quality control and quality assurance protocols were followed (QA/QC) to meet the 1988 Clinical Laboratory Improvement Act mandates.

### 2.9. Statistical Analysis

Normality and homogeneity of the relative reproductive index, reproductive biomarkers, and hormonal content were statistically analyzed by following one-way analysis of variance (ANOVA) and Tukey-Dunnett test using SPSS 16.0 to determine the significant variations between the control and TBT treated groups. As no considerable variations between negative and solvent control have been noticed, only the negative control has been taken for statistical comparison.

## 3. Results

### 3.1. Assessment of Reproductive Activity

**Figure 1:**
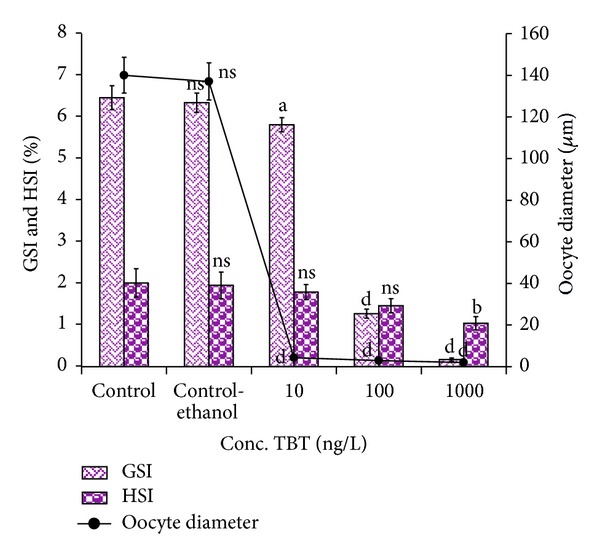
Effect of TBT on the GSI, HSI, and oocyte growth in* M. rosenbergii.* Each value is a Mean ± SD of three replicates. ns: non significance; a: *P* < 0.05; b: *P* < 0.01; c: *P* < 0.001; d: *P* < 0.0001.

TBT had significantly reduced the GSI and HSI values in TBT treated prawns. In control, the GSI and HSI values were recorded as 6.46 ± 0.29% and 2.01 ± 0.34%, respectively. The GSI and HSI values steadily declined in exposed prawns as the concentration of TBT increases. At higher concentration of 1000 ng/L TBT, the GSI and HSI values were decreased drastically to 0.17 ± 0.04% and 1.04 ± 0.16%, respectively, after  6 months of exposure (Figure 1). The GSI and HSI values were statistically varied between control and TBT treated groups (*P* > 0.05 to *P* < 0.001).

### 3.2. Morphological Alterations of Ovary and Hepatopancreas

**Figure 2:**
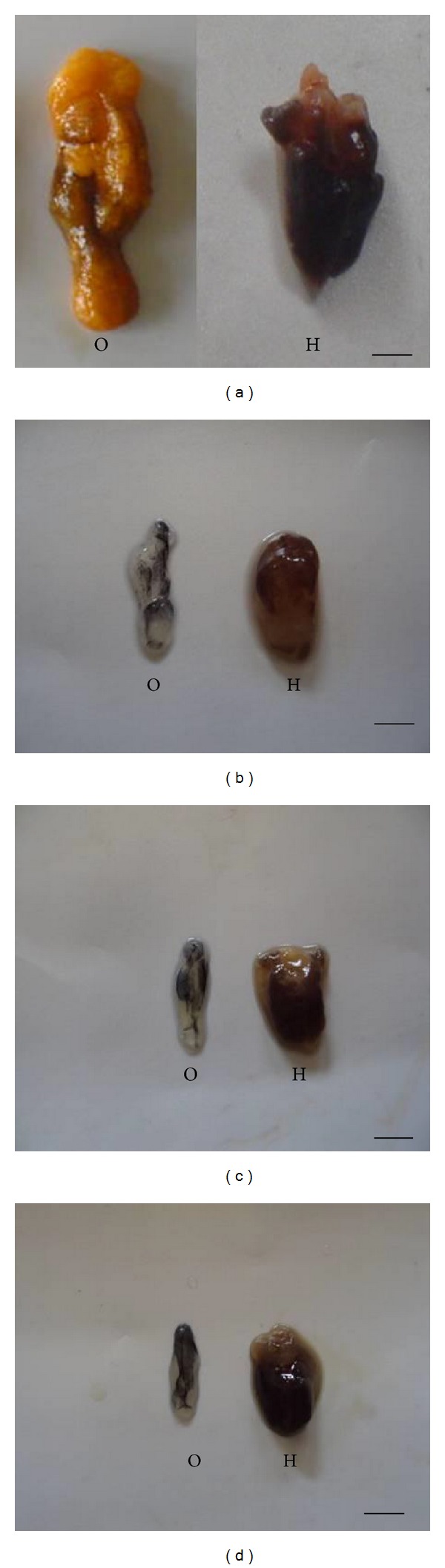
(a) Control prawn showing fully mature vitellogenic stage ovary (O) and hepatopancreas (H). (b), (c), (d) Showing variation in the gross morphology of ovary and hepatopancreas in TBT treated prawns. (b) Note the reduction in the ovarian development at spent stage at 10 ng/L, (c) spent stage ovary at 100 ng/L, and (d) no ovarian development at 1000 ng/L. Note the decrease of ovarian development and variation in size of hepatopancreas in TBT treated prawns compared to control.

TBT influenced the morphological structure of the ovary as well as of the hepatopancreas. Control prawns showed a fully mature ovary in the vitellogenic stage (Figure 2(a)). At 10, 100, and 1000 ng/L of TBT exposure, the ovarian development was arrested during spent stage in prawns (Figures 2(b)–2(d)) and also the size of the hepatopancreas was found to be reduced in all exposure groups compared to control.

### 3.3. Cellular Level Changes in Ovary

**Figure 3:**
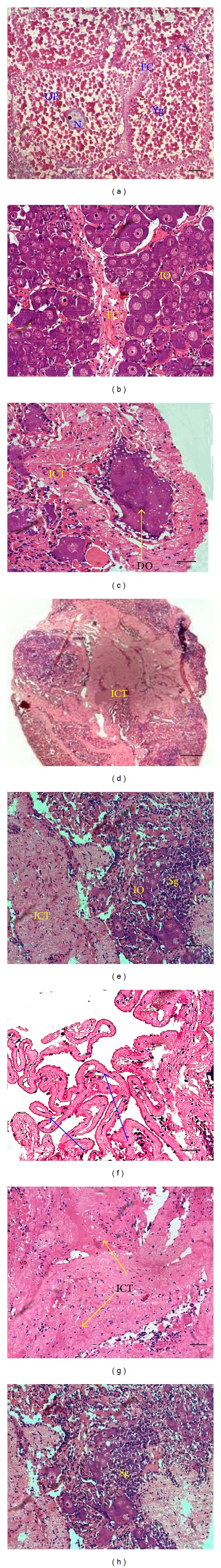
(a) Cross-section through ovary of prawn (control) showing vitellogenic oocytes with distinct ooplasm (OP) filled with yolk globules (Yg) with nucleus (N). Each oocyte is enveloped by a row of characteristic follicle cells (FC). (b) At 10 ng/L TBT exposure, ovary showing previtellogenic oocytes (IO) and follicle cells (FC). (c) Ovary showing the fusion of developing oocytes (↑DO) and increase in interstitial connective tissues (ICT) at 100 ng/L TBT exposure and (d) At 1000 ng/L TBT exposure, ovary showing immature oocytes and increase in interstitial connective tissues (ICT). (e) Higher magnification of the above, showing ovotestis formation with spermatogonia (Sg), increase in interstitial connective tissues (ICT), and immature oocytes (IO). (f) And (g) ovary showing connective tissues modified into tubular like structure (↑) and increase in interstitial connective tissues (↑ICT). (h) Ovary of prawn treated with TBT (1000 mg/L) showing occurrence of spermatogonia (Sg) in the ovary. Bar: 50 *μ*m.

Control prawns showed normal development with vitellogenic oocytes containing distinct ooplasm filled with yolk globules. The oocytes were enveloped by a row of characteristic follicle cells with prominent nucleus and nucleolus (Figure 3(a)). At 10 ng/L of TBT, the ovary exhibited immature oocytes, reduction in the size of the oocyte, and absence of yolk material (Figure 3(b)). Fusion of the developing oocytes and increase in the interstitial connective tissues were noticed in the ovary at 100 ng/L TBT exposure (Figure 3(c)). At 1000 ng/L TBT an ovotestis formation with numerous spermatocytes as well as interstitial connective tissue of tubular nature was found (Figures 3(d)–3(f)).

### 3.4. Oocyte Growth

TBT also affected the oogenesis as evidenced by the decrease in oocyte diameters. The oocyte size of the ovaries was decreased in all the treated groups. The oocyte diameter of control prawn measured 140 ± 8.57 *μ*m. At 10 ng/L the oocyte diameter was 4.26 ± 0.76 *μ*m and at 1000 ng/L it was reduced to 1.91 ± 0.47 *μ*m. Overall, the oocyte diameter decreased with an increase in TBT concentration (Figure 1). Tukey-Dunnett test revealed that the oocyte diameter was statistically differed (*P* > 0.05 to *P* < 0.001).

### 3.5. Ultrastructural Changes in Ovary

**Figure 4:**
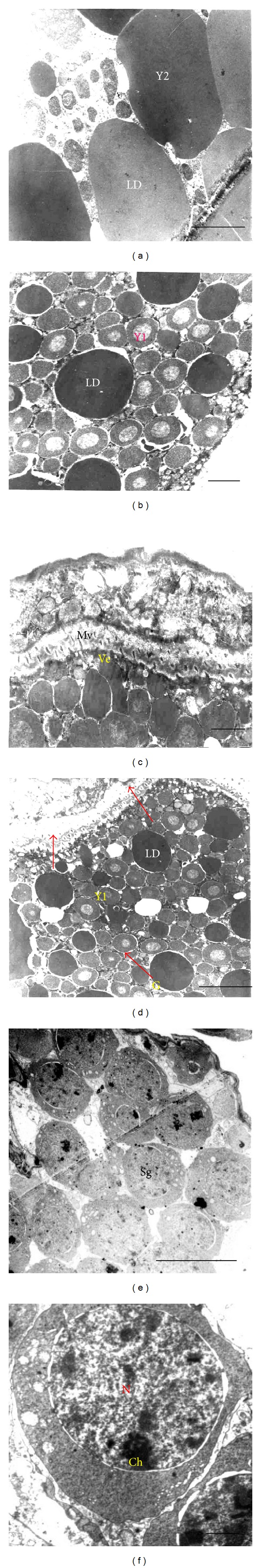
(a) Transmission electron micrograph (TEM) of section through the ovary (control) showing mature oocytes with type 2 yolk globules (Y2) and lipid droplets (LD) (×4,500). Bar: 1 *μ*m. (b) TEM of section through the ovary of TBT treated prawn showing type 1 yolk globules (Y1) and lipid droplets (LD) (×7,000). Bar: 1 *μ*m. (c) TEM of control prawn ovary showing normal architecture of vitellin envelop (Ve) and uniformly arranged microvilli (Mv) (×10,000). Bar: 1 *μ*m. (d) TEM of section through ovary of the treated prawn showing oocytes with type 1 yolk globules (Y1), lipid droplets (LD), glycogen particles (↑G), and reduced size of microvilli (↑) (×4,500). Bar: 1 *μ*m. (e) TEM of section through the ovary showing the spermatogonia (Sg) in the treated prawn (×1,500). Bar: 10 *μ*m. (f) Enlarged view of spermatogonia with centrally located nucleus (N) and chromatin (Ch) distributed throughout the nucleoplasm (×10,000). Bar: 1 *μ*m.

Transmission electron micrograph of control ovary (late vitellogenic stage) showed normal architecture of oocytes with ooplasm filled with prominent type 2 yolk globules and lipid droplets. The other cellular organellae such as vitellin envelop and microvilli were regularly arranged in the control prawn (Figures 4(a), 4(c), and 4(e)). In TBT treated prawn, the ovary displayed type 1 yolk globules, glycogen particles, and reduction in length of microvilli. On the other hand, spermatogonial cells are also present in the ovary. Nucleus is located in the centre of spermatogonial cells (Figures 4(b), 4(d), and 4(f)).

### 3.6. Vitellin Content in Ovary

**Figure 5:**
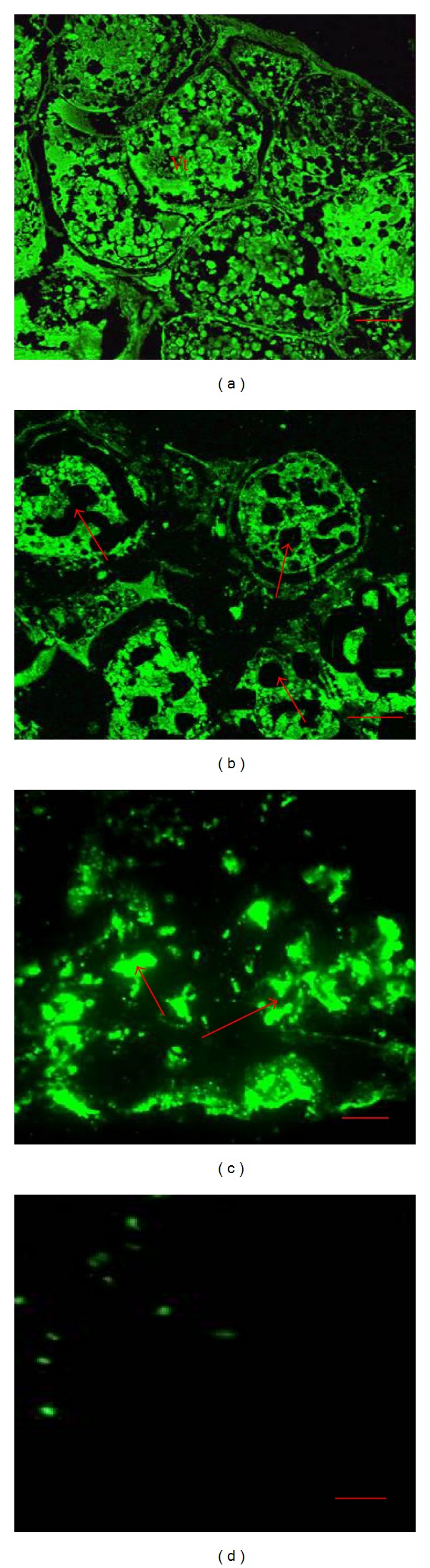
Immunofluorescence expression of vitellin in ovary. (a) Control prawn ovary showed maximum immunostaining expression of vitellin (Vt). (b) Reduction of vitellin (↑) as evident with less intensity of immunostaining at 10 ng/L exposure. (c) Less expression of vitellin (↑) as well as fusion of vitellin content as indicated with moderate immunostaining at 100 ng/L exposure. (d) At 1000 ng/L TBT, treated ovary showing absence of the vitellin. Note the reduction in the intensity of the vitellin in the ovary of treated prawns. Bar: 50 *μ*m.

The immunofluorescence staining clearly indicated the high vitellin content in control prawns by green fluorescence (Figure 5(a)). At 10 ng/L treatment, the ovary exhibited less immunostaining as a direct evidence of reduction in vitellin content (Figure 5(b)). At 100 mg/L TBT, the ovary showed the least amount of vitellin content as well as fusion of the vitellin content (Figure 5(c)). On the other hand, at 1000 ng/L TBT treated ovary, no immunostaining was observed as a result of absence of the vitellin content (Figure 5(d)).

### 3.7. Quantification of Biomarkers of Vitellogenesis

**Figure 6:**
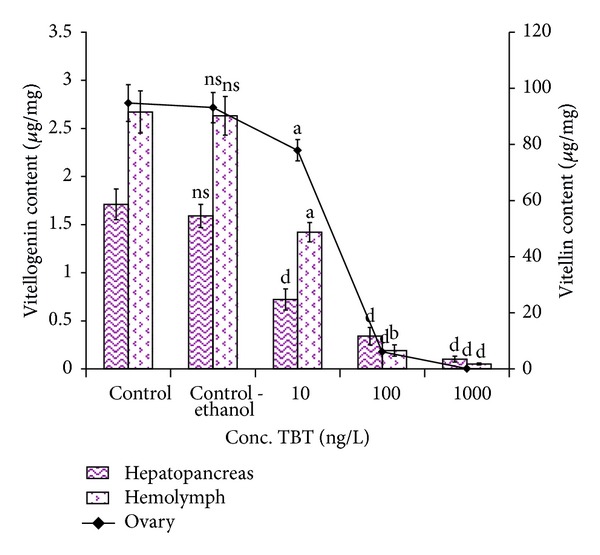
Impact of TBT on the vitellogenin and vitellin content in* M*.* rosenbergii*. Each value is a Mean ± SD of three replicates. ns: non significance; a: *P* < 0.05; b: *P* < 0.01; c: *P* < 0.001; d: *P* < 0.0001.

The results clearly indicated that vitellogenin and vitellin content decreased significantly due to the exposure of TBT, compared to control. In control prawn, the vitellogenin content in hepatopancreas and hemolymph was recorded as 1.71 ± 0.16 *μ*g/g and 2.67 ± 0.22 *μ*g/mL, respectively. Interestingly, at higher concentration of TBT (1000 ng/L), vitellogenin content reduced drastically in both hepatopancreas (0.10 ± 0.03 *μ*g/g) and hemolymph (0.05 ± 0.01 *μ*g/mL) after six months of exposure. On the other hand, vitellin content was notably decreased to 77.9 ± 3.77 *μ*g/g and 0.11 ± 0.02 *μ*g/g in 10 ng/L and 1000 ng/L of TBT treated groups, respectively (Figure 6). However, the vitellin content of control ovary was 94.71 ± 6.55 *μ*g/g. The variation of vitellogenin and vitellin content in TBT treated groups differed significantly from that of control group (*P* > 0.05 to *P* < 0.0001).

### 3.8. Quantification of Sex Hormones

#### 3.8.1. 17*β*-Estradiol Levels

**Figure 7:**
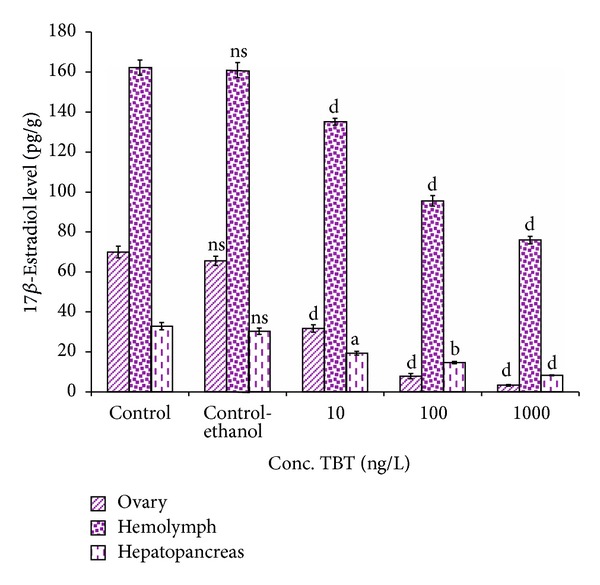
Impact of TBT on the 17*β*-estradiol in different reproductive tissues in* M. rosenbergii.* Each value is a Mean ± SD of three replicates. ns: non significance; a: *P* < 0.05; b: *P* < 0.01; c: *P* < 0.001; d: *P* < 0.0001.

In TBT treated prawns, 17*β*-estradiol level decreased significantly compared to control (Figure 7). In control prawn, 17*β*-estradiol levels in ovary, hemolymph, and hepatopancreas were recorded as 69.9 ± 2.86 pg/g, 162.2 ± 3.76 pg/mL, and 32.8 ± 1.80 pg/g, respectively. On exposure to TBT (10 ng/L), 17*β*-estradiol level reduced to 31.7 ± 1.80 pg/g in ovary, 135.1 ± 1.71 pg/mL in hemolymph, and 19.3 ± 0.98 pg/g in hepatopancreas. However, at higher concentration of TBT (1000 ng/L), 17*β*-estradiol level decreased drastically in ovary (3.3 ± 0.41 pg/g), hemolymph (75.9 ± 1.88 pg/mL), and hepatopancreas (8.2 ± 0.24 pg/g). The decrease of 17*β*-estradiol levels in control and TBT treated groups at 100 and 1000 ng/L was significant (*P* < 0.05). Estradiol level in ovary, hemolymph, and hepatopancreas was statistically varied (*P* > 0.05  to  *P* < 0.001) between control and experimental groups.

#### 3.8.2. Testosterone Level in Ovary

**Figure 8:**
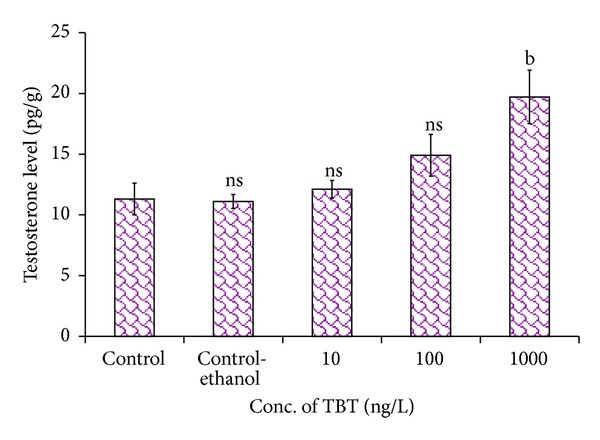
Impact of TBT on the testosterone in ovary of* M. rosenbergii.* Each value is a Mean ± SD of three replicates. ns: non significance; a: *P* < 0.05; b: *P* < 0.01; c: *P* < 0.001; d: *P* < 0.0001.

The level of testosterone gradually increased in the ovary of TBT treated groups (Figure 8). The testosterone level in the ovary of control prawns was recorded as 11.3 ± 1.31 pg/g. The testosterone level showed a marginal increase of 12.1 ± 0.73 pg/g at 10 ng/L and 14.9 ± 1.71 pg/g at 100 ng/L. At higher concentration of TBT (1000 ng/L), the testosterone level increased to 19.7 ± 2.20 pg/g in the ovary. The changes in testosterone levels were varied significantly in the TBT treated and control groups (*P* > 0.05  to  *P* < 0.01).

## 4. Discussion

Xenobiotics are known to affect aquatic organisms at all stages of their reproductive cycle like gametogenesis, fertilization, maturation, spawning, embryonic development, and sex differentiation [10]. There is an increasing substantiation that many xenobiotic chemicals reduce the reproductive capacity of the aquatic animals through disruption of vitellogenesis [38]. Moulting and reproduction are the two major physiological processes demanding energy in crustaceans [39].

Our results clearly demonstrated that TBT had considerably reduced the oogenesis as evident with the measurements of GSI, HSI and oocyte diameter compared to control. Accordingly, Zhang et al. [33] suggested that TBT can affect the GSI and HSI in female cuvier* Sebastiscus marmoratus*. The higher concentration of TBT arrested the ovarian development on spent stage. Besides, TBT also induced the ovotestis formation in* M. rosenbergii*. The results revealed the dose dependent toxicity of TBT on the reproductive system of* M. rosenbergii. *Similarly, Rodríguez et al. [40] reported the impaired development of ovaries in fiddler crab* Uca pugilator* due to TBT toxicity.

Histopathological and ultrastructural studies are necessary for the description and evaluation of potential lesions in aquatic animals exposed to various toxicants [41]. Ovarian development in TBT treated prawns indicated the reduction in oocyte diameter, yolk globules, fusion of immature oocytes, and disruption of follicle cells. Besides, the ultrastructural changes are prominent in the ovary of* M. rosenbergii*, exposed to TBT. Histoanatomical abnormalities in the ovaries may be caused by xenobiotic toxicants [42] and effluent and aquatic pollutants [43–48]. Giri et al. [49] reported the effects of insecticide basathrin induced anatomical changes in the ovary of catfish,* Heteropneustes fossilis*. They reported marked damage in germinal epithelium, atresia of oocyte, stromal hemorrhage, vacuolization of oocytes, and general inflammation.

The present study reveals the ovotestis formation in female prawn* M. rosenbergii *due tothe exposure of TBT. At higher concentration of TBT, the ovary showed immature oocytes as well as spermatogonial cells, increased interstitial connective tissues, and tubule-like structure formation in the ovary. Likewise, Oehlmann et al. [50] reported the gonadal tissues of ovaries with a small amount of the testis that was observed. This is basically similar to the imposex in meso- and neogastropods, which is known to be typically induced by organotion compounds, such as TBT and triphenyltin (TphT) from antifouling paints. In this connection, TBT has classically been reported to be a strong EDC, inducing masculinization of gastropod females probably by interfering with more than one mechanism, that is, by inhibiting P450-dependent aromatase that converts endogenous testosterone to estradiol, as well as by inhibiting testosterone excretion, therefore giving rise to a phenotype known as imposex [19]. Besides, TBT can also alter the sex ratio towards males in zebrafish* Danio rerio* [51, 52]. In addition, Nakayama et al. [53] found that TBT also reduced the reproductive frequency of medaka fish. The molluscs collected from TBT polluted sites were observed to be masculinized. Thus, organotin compounds especially TBT cause the intersex and reproductive failure in gastropods.

Variation in the levels of Vg and Vt can provide a useful marker of dysfunction caused by xenobiotics in the reproductive axis [54]. The present study clearly documents the impact of TBT on the vitellogenesis by drastically reducing the vitellogenin and vitellin content. Low concentration and absence of Vg and Vt are an indication of a malfunction resulting in inhibited ovarian growth and could affect the reproductive activity of* M. rosenbergii*. Studies on the biochemical changes in the ovary and other organ systems during the active vitellogenesis are very important since the ovary starts accumulating the yolk protein, vitellin. The protein is metabolized to produce glucose by the process of gluconeogenesis and it is utilized for energy production during stressful condition [55]. The reduction in vitellogenin content influenced by naphthalene stress may be attributed to the utilization of amino acids in various catabolic reactions. The amino acids through transamination and deamination actions might have supplied indispensable ketoacids to act as precursors for the maintenance of carbohydrate metabolism to meet the energy requirements during pollutant exposure [56]. The increase in amino acid content of the hepatopancreas, hemolymph, and ovary of* Scylla serrata* reared in naphthalene medium might be due to proteolysis process to meet the energy demand of crabs under naphthalene stress and is consistent with decreased protein content in the ovaries of TBT treated prawns [57].

The present study clearly indicates that the reduction in the level of GSI can therefore be a marker of TBT induced dysfunction during vitellogenesis. Therefore, due to the exposure of TBT, the lower level of Vg and Vt content during ovarian maturation was noticed as an indication of malformation at another point of the reproductive endocrine system. In this context, the inhibition in ovarian development as well as ovotestis formation in* M. rosenbergii* was observed. In consonance, the reduction of Vg and Vt contents due to the blocking of protein synthesis or protein denaturation or interruption in the amino acid synthesis ascribable to naphthalene toxicity was documented by Vijayavel and Balasubramanian [55]. The depletion may also be due to the rapid utilization of protein by the cells under stress condition and also indicates that the protein might undergo proteolysis which results in the production of free amino acids and is used for energy production during stress condition [56]. The alterations in the GSI can be used as a hazard criterion for assessing the reproductive fitness or competence in all forms of oviparous animals [58]. The decline in the Vg and Vt content might have influenced the reduction of GSI and the rate of egg production, and finally thereby affecting population of* S. serrata* by naphthalene exposure [59]. Thus, it is clear that many xenobiotic chemicals including naphthalene can disturb vitellogenesis by the direct action of the toxicants [54].

Sex hormones derived from the gonads play crucial roles in sexual differentiation, maturation, and behaviour in vertebrates [53]. It is well know that 17*β*-estradiol, once secreted into the circulation, stimulates the hepatic production of vitellogenin, necessary for oocyte maturation [60]. In the ovary, 17*β*-estradiol is catalyzed by the steroid synthesizing enzymes, in particular the P450 aromatase. In teleosts, changes in the activity and expression of the P450 aromatase have been shown to drive changes in the ovarian production of 17*β*-estradiol during the reproductive cycle [61].

The present study exhibited the increase in the levels of testosterone and decreased levels of 17*β*-estradiol in the ovary of prawns exposed to TBT, which could be associated with an inhibition with P450 aromatase activity. Thus, the changes of sex hormone levels would finally influence the ovarian development and ovotestis formation in prawn. Zhang et al. [33] reported similar changes in ovarian development as well as sex hormonal changes in cuvier,* Sebastiscus marmoratus.* The TBT also had possible effects on the reproductive activity of the crab* Clibanarius vittatus* [62]. The present study explains the masculinizing effect, such as imposex, bias of sex towards males in prawns imputable to TBT. Despite this, TBT has also induced hermaphroditism in* M. rosenbergii*. Supportively, Shimasaki et al. [63] found that the TBT has potentially induced masculinization to the extent of complete sex reversal in the genetically female Japanese flounder* Paralichthys olivaceus*.

The hormonal balance between estradiol and testosterone appears to be crucial in development of gametogenesis in crustaceans [64]. This balance relies on the availability and activity of the steroid-synthesizing enzymes and in particular on the cytochrome P450 aromatase that catalyzed estradiol from the conversion of aromatizable testosterone. It has been suggested that the reproductive toxicity of organotin compounds is due to inhibition of the aromatase [64]. The masculinizing effect of TBT in prawn may also inhibit the aromatase activity. The present study showed an increase in testosterone levels and a decrease in 17*β*-estradiol levels in female prawn,* M. rosenbergii, *which were also due to TBT exposure.

## 5. Conclusion

The present investigation provides ample evidence that the TBT has significantly affected the reproductive activity of* M. rosenbergii* by means of imbalance in sex hormones such as decrease in 17*β*-estradiol and increase in testosterone levels ascribable to endocrine dysfunction. The above phenomenon that led to ovotestis formation in the TBT exposed female prawn* M. rosenbergii *was noticed. Out of the present study, we inferred that the TBT induces masculinization of female prawns into male prawns, though the mechanisms behind the TBT induced endocrine dysfunction yet to be revealed with receptor complex and gene expression studies.
